# Role of Oral Nutritional Supplements in Minimizing the Risk of Postoperative Malnutrition in Patients Undergoing Gastrointestinal Surgery

**DOI:** 10.3390/jcm15124587

**Published:** 2026-06-12

**Authors:** Jarosław Cwaliński, Adam Bobkiewicz, Agnieszka Cwalińska, Wiktoria Zasada, Hanna Cholerzyńska, Tomasz Banasiewicz, Barbara Kuczyńska

**Affiliations:** 1 Department of General, Endocrinological Surgery and Gastroenterological Oncology, Poznan University of Medical Sciences, 60-355 Poznan, Poland; 2 Department of Infectious Diseases and Child Neurology, Poznan University of Medical Sciences, 61-701 Poznan, Poland

**Keywords:** prehabilitation, malnutrition, ERAS, oral nutritional supplementation, perioperative nutrition

## Abstract

**Background/Objectives**: Perioperative fasting combined with procedure-related trauma increases the risk of malnutrition and determines the treatment outcomes of surgical patients. The aim of this study was to assess the range of metabolic deficiencies and the effectiveness of oral nutritional supplements (ONSs) after surgical intervention. **Methods:** 84 patients undergoing elective abdominal surgery were included in this study. Patients were divided into three groups: receiving a low-osmotic ONS (Group I), high-osmotic ONS (Group II), and Group III, who did not receive any oral supplementation. The clinical assessment involved body weight measurements and metabolic blood tests preoperatively and on the 4th, 7th, 14th, and 28th postoperative days. **Results:** The mean blood levels of total protein, albumin, cholesterol, and blood lymphocyte count decreased in the first 4 days after surgery and returned to baseline between 7 and 14 days. Similarly, BMI dropped during the first two weeks and then stabilized or returned to pre-surgery values. Triglycerides initially increased and, after 14 days, started to normalize. Patients receiving an ONS compensated quicker than the control group and more efficiently with low-osmotic supplements. **Conclusions:** Surgical trauma is associated with metabolic deficiency. The early administration of ONSs provides significant benefits to patients undergoing abdominal surgery. Low-osmotic supplements are especially recommended due to better tolerance.

## 1. Introduction

Effective perioperative management with a particular focus on secondary malnutrition is a cornerstone of advanced surgery. It holds an equal priority alongside technical innovations, operator training, and the experience of the therapeutic team [1]. For over 30 years, clinical evidence has consistently demonstrated that the optimization of patients prior to elective surgeries through nutritional support, improved physical fitness, and functional capacity, as well as a cessation of smoking and alcohol consumption, have a positive effect on the outcomes of surgical management [2,3,4].

An increasing body of literature emphasizes the importance of optimal patient preparation before surgery. This preparation not only aims to minimize perioperative complications but also enhances long-term outcomes and shortens hospital stays. In the long term, individualized perioperative care standards contribute to improved quality of life and help mitigate the rising healthcare costs associated with uncontrolled hospitalization [5,6,7].

At present, preventing the risk of perioperative malnutrition is a central focus of prehabilitation protocols, serving as a crucial determinant in achieving early recovery after surgery, as outlined by the Enhanced Recovery After Surgery (ERAS) approach. Research indicates that 10 to 65% of surgical patients either require preoperative nutritional support or are at risk of malnutrition prior to surgery [8,9,10]. The European Society of Parenteral and Enteral Nutrition (ESPEN) recommends at least 7-day comprehensive nutritional treatment before surgery [11]. Furthermore, reducing the fasting duration and selecting appropriate postoperative dietary regimens not only accelerate recovery but also reduce the hospital stay, resulting in lower overall healthcare costs [11,12].

In accordance with ERAS protocol and ESPEN guidelines, individuals identified as malnourished or at risk of malnutrition should receive oral nutritional supplements (ONS) as a fundamental part of their perioperative care [1,13].

However, ONSs differ in composition, primarily in the amount of carbohydrates and protein, which determines their caloric value and osmolality. Hence, in clinical practice, nutritional products are divided into dietarily complete and incomplete, depending on whether the selection and amount of nutrients are intended to reflect a balanced meal or only selectively enrich the diet [14]. However, even a multi-ingredient product compiled strictly according to the patient’s needs is not always effective in postoperative nutrition. The complete ONSs, despite their appropriate composition, are in practice less well tolerated by patients, especially in the first days after the procedure. This inevitably raises the question of whether ONSs of different composition and osmolality modulate the metabolic condition of surgical patients [15].

The aim of this study is to evaluate the impact of complete and incomplete oral nutritional supplements on reducing postoperative malnutrition in patients undergoing abdominal surgery.

## 2. Materials and Methods

The study was designed as a prospective observational study involving patients undergoing abdominal surgery in the authors’ department from 2019 to 2024. Nutritional support in surgical patients was established in accordance with the European Society of Parenteral and Enteral Nutrition (ESPEN) [16] and the Global Leadership Initiative on Malnutrition (GLIM) [17] recommendations, which define nutritional therapy as the possibility of providing nutrients orally through ONS administration in order to reduce the risk of malnutrition associated with perioperative starvation and lack of food. Screening for malnutrition was performed based on phenotypic and etiologic cryotherapy, considering surgical trauma (inflammation) and non-volitional weight loss immediately after surgery as the basic eligibility criteria. To standardize the criteria related to the surgical risk and the extent of the procedure, the study included patients only undergoing elective surgery, with a surgical complexity of grade 3 according to the National Institute for Health and Care Excellence (NICE) classification [18] and with anesthesia risk grades I and II, according to the American Society of Anesthesiologists (ASA) score.

The qualification process consisted of three stages. (1) Pre-elimination: patients were evaluated to ensure they met the inclusion criteria for surgical treatment and nutritional support, with simultaneous verification of the absence of preoperative exclusion factors; (2) protocol enrollment: eligible patients were assigned to different postoperative nutritional supplementation strategies; and (3) post-study exclusion: patients who experienced postoperative complications that could significantly impact their metabolic status or the final treatment outcomes were excluded from the study.

All participants included in the analysis met the following criteria: (1) individuals 18 years of age or older; (2) at least one segmental resection of the small and/or large intestine with at least one intestinal anastomosis; (3) laparotomy approach; (4) in case of colorectal cancer—tumors no more locally advanced than T2 according to the TNM classification; (5) BMI ≥ 20, with no clinical or laboratory signs of malnutrition; (6) Nutritional Risk Screening 2002 (NRS 2002) score less than 3; and (7) informed consent for participation in the study. As part of the standard procedure, the continuity of the gastrointestinal tract was evaluated preoperatively through radiological and/or endoscopic examinations. Additionally, the preservation of oral feeding capability was ensured in all cases.

Patients were excluded from the study if any of the following conditions were present: (1) postoperative obstruction of the gastrointestinal tract; (2) complications requiring relaparotomy (3) psychiatric eating disorders; (4) BMI < 20; (5) chronic or acute liver failure; (6) chronic or acute kidney disease; (7) persistent vomiting and/or diarrhea requiring antidiarrheal; (8) short bowel syndrome diagnosed preoperatively or revealed postoperatively; (9) allergy to any ingredient of the ONS; (10) uncontrolled diabetes; (11) hospital stay in an intensive care unit < 1 day; and (12) failure to adhere fully to the prescribed ONS regimen.

**Figure 1:**
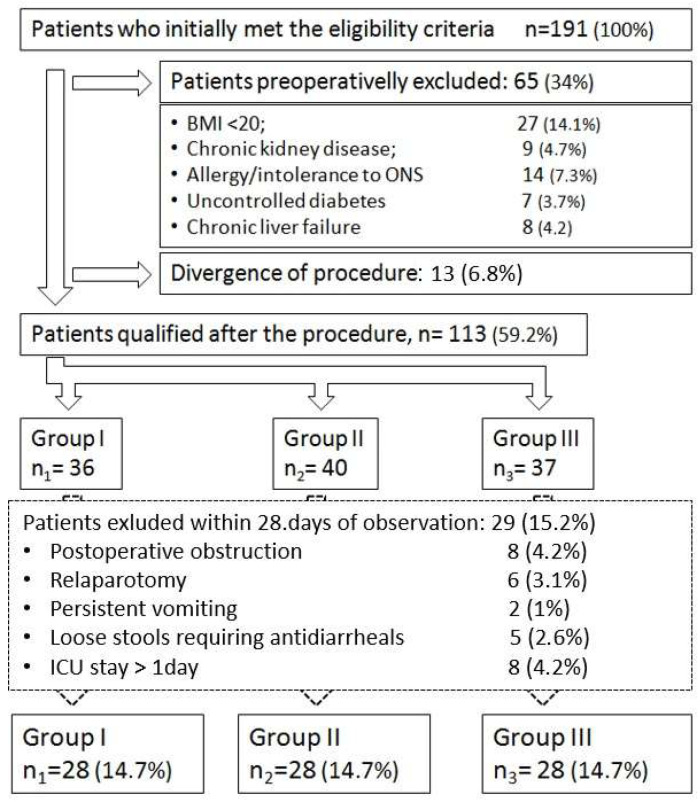
The flowchart presenting the qualification scheme for the study, with a division of reasons for exclusion before and after surgical procedure.

Consecutive patients who met the inclusion criteria and simultaneously did not have any exclusions were qualified for the study. The enrollment was completed in December 2023, ensuring an equal number of patients in all three groups. In accordance with the eligibility criteria, 191 patients were initially qualified for the study, with 84 remaining for the final evaluation. Recruitment details are shown in the study flowchart (Figure 1).

In this study, we utilized a per-protocol approach to ensure the reliability of our findings. Only patients who fully adhered to the intervention protocol were included in the final analysis. Adherence was closely monitored by the research team through direct observation and regular documentation of ONS intake. Patients were required to consume the prescribed ONS formulations as specified in the study protocol, and any deviations, such as incomplete consumption or non-compliance, resulted in exclusion from the per-protocol analysis.

To clarify the observations before and after the procedure, we have defined the timeline of events relative to the day of surgery as follows: the day preceding the surgery was referred to as Day −1. The days following the surgery were described as Day 1, Day 2, Day 3, and so on until the end of surveillance on Day 28.

### 2.1. Study Design

All patients included in the study received clear fluids orally, supplemented with commercially available ready-to-use parenteral nutrition on the first postoperative day. Starting from the second or third day onwards, a full liquid diet consisting of soups and pulps was introduced, gradually progressing to solid food by the fourth postoperative day.

Based on the receipt of oral nutritional supplementation, eligible patients were evaluated according to the following protocol: (1) Group I (*n* = 28)—patients received a low-osmotic ONS formula (Nutramil Complex Protein, Olimp Labs, Pustynia, Poland), administered at a dose of 200 mL twice daily, starting from the first postoperative day and continuing until the fourteenth day postoperatively; (2) Group II (*n* = 28)—patients received a high-osmotic ONS formula (Nutridrink Protein, Nutricia Polska, Warszawa, Poland), administered at a dose of 200 mL twice daily, starting from the first postoperative day and continuing until the fourteenth day postoperatively; and (3) Group III (*n* = 28)—served as a control group and did not receive any ONS postoperatively. The characteristics of the ONS formulas used in the study are summarized in Table 1.

### 2.2. Methods of Assessment

Clinical evaluation included BMI measurement taken on the day prior to surgery, as well as on the 14th and 28th days postoperatively. The metabolic effects of the nutritional intervention were assessed using a blood test panel on the day before the surgery and postoperatively on the 4th, 7th, and 14th days. The timeframe and parameters evaluated in the study are summarized in Table 2.

### 2.3. Ethical Statement

The therapy described in this study adhered to the principles of medical ethics and good medical practice. In accordance with the Bioethics Committee at the Poznan University of Medical Sciences, the study’s methods and assumptions did not require individual consideration by the Bioethics Committee. This study was conducted in accordance with the principles of the Declaration of Helsinki.

### 2.4. Statistical Analysis

Statistical analysis was performed using Statistica software (Statsoft version 6.0). The results are presented as mean values ± standard deviation (SD). Statistical analysis of matched variables for more than two study groups was performed using analysis of variance (ANOVA) with Friedman’s modification (for nonparametric variables) and Dunn’s post hoc test (for parametric variables). For unpaired nonparametric data, analysis of variance (ANOVA) with the Kruskal–Wallis modification was used. Data compared between two groups were evaluated using Student’s *t*-test and Fisher’s exact test. A *p*-value of 0.05 was considered statistically significant.

## 3. Results

The study population included 84 patients, with 52 (61.9%) females and 32 (38.1%) males. The mean age at the time of surgery was 59 years (range: 23–85 years). The basic characteristics of the patient cohort are presented in Table 3.

The most common indicators for surgery were colorectal cancer localized to the colon (*n* = 30) and rectum (*n* = 21). Non-oncological surgery was performed in 29 patients, with the restoration of the GI tract continuity (reversal of Hartmann’s procedure) and complicated diverticular disease as the most common indicators. Detailed characteristics of the underlying pathologies are summarized in Table 4. In the majority of cases (*n* = 80), a single intestinal anastomosis was performed, while four patients required two anastomoses.

All surgeries were performed using a laparotomy approach with a midline incision. A stapled technique was used in 67 patients, and hand anastomoses were performed in 17 patients. Circular staplers and linear staplers were used in 44 and 23 patients, respectively. The characteristics of surgical management are summarized in Table 4. The mean duration of surgery was 170 min (range: 75–230 min).

**Figure 2:**
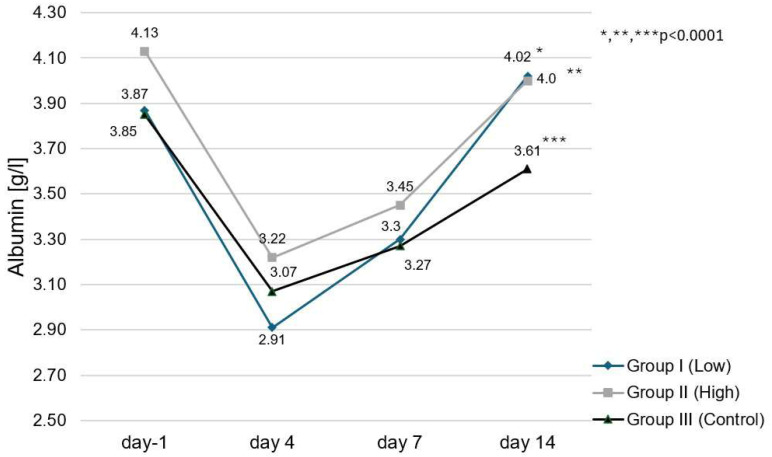
Alterations in serum albumin level in the group of patients taking low-osmotic (Group I) and high-osmotic (Group II) oral nutritional supplements in relation to the control group (Group III). The standard deviation (SD) did not exceed 0.71 in Group I, 0.37 in Group II, and 0.54 in Group III (not plotted).

**Figure 3:**
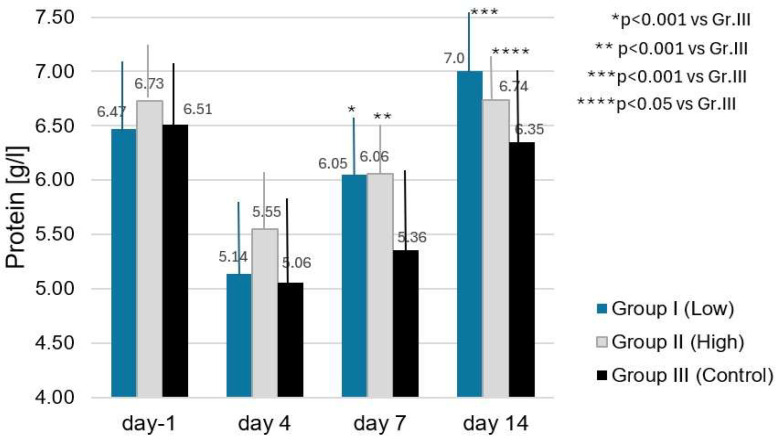
The total protein concentration in the group of patients taking low-osmotic (Group I) and high-osmotic (Group II) oral nutritional supplements in relation to the control group (Group III) measured before and after the procedure.

**Figure 4:**
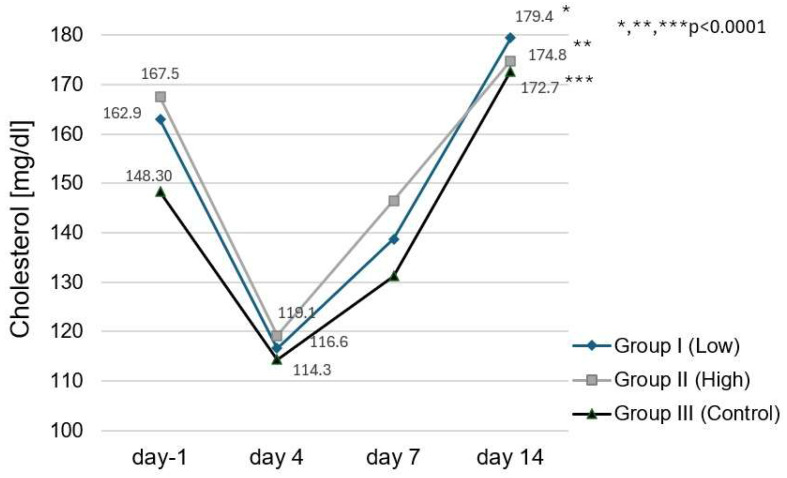
Perioperative changes in the serum cholesterol level observed in the group of patients taking low-osmotic (Group I) and high-osmotic (Group II) oral nutritional supplements and the control group (Group III). The standard deviation (SD) did not exceed 40.9 in Group I, 36.4 in Group II, and 29.1 in Group III (not plotted).

**Figure 5:**
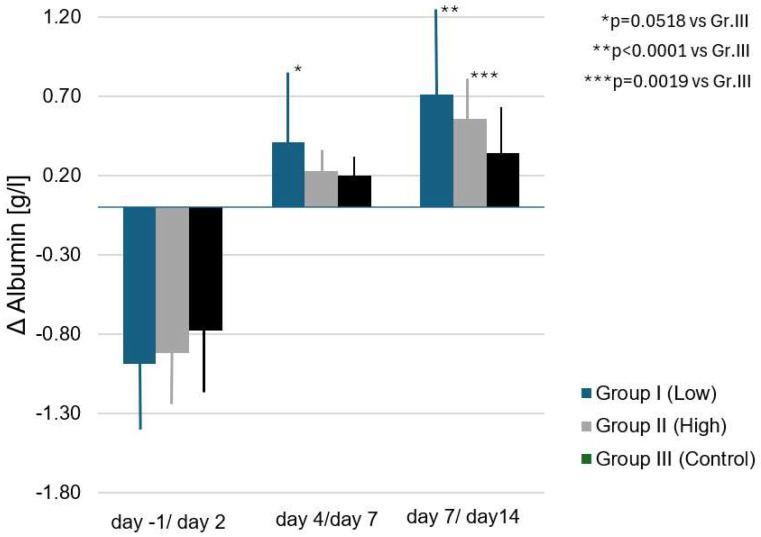
Relative changes in the mean albumin level (Δ albumin) measured from the day before surgery (Day −1) to Day 2 after the procedure, then from postoperative Day 4 to 7 and from Day 7 to 14 in patients receiving a low-osmotic (Group I) or high-osmotic (Group II) oral nutritional supplement in relation to the control group (Group III).

**Figure 6:**
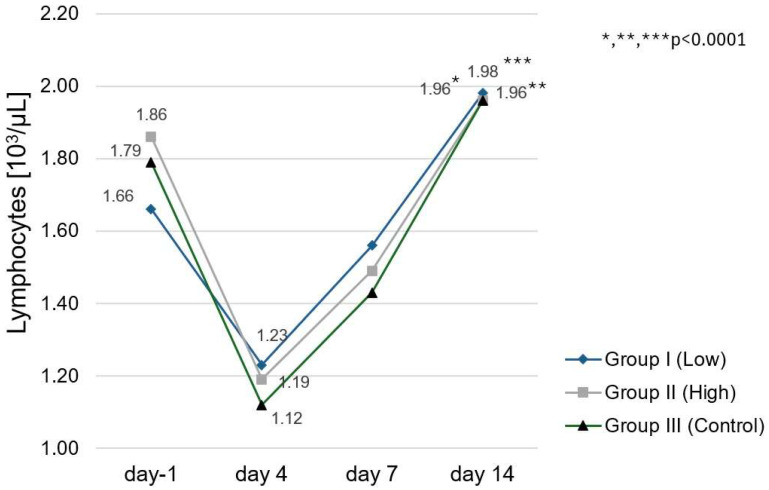
The level of lymphocytes in blood serum in the group of patients taking low-osmotic (Group I) and high-osmotic (Group II) oral nutritional supplements in relation to the control group (Group III). The standard deviation (SD) did not exceed 0.63 in Group I, 0.33 in Group II, and 0.54 in Group III (not plotted).

In all study groups, albumin, total protein, and total cholesterol levels rapidly decreased by the 4th postoperative day. An increase in these parameters was observed on the 7th and 14th postoperative days. Notably, only in Groups I and II did the final protein and albumin levels surpass the baseline values measured prior to surgery (Figure 2, Figure 3 and Figure 4). Patients receiving the low-osmotic ONS had the highest albumin concentration at the beginning of the second week post-surgery (Figure 5). Similar trends were observed in lymphocyte levels, though no statistically significant differences were found between the study groups (Figure 6).

**Figure 7:**
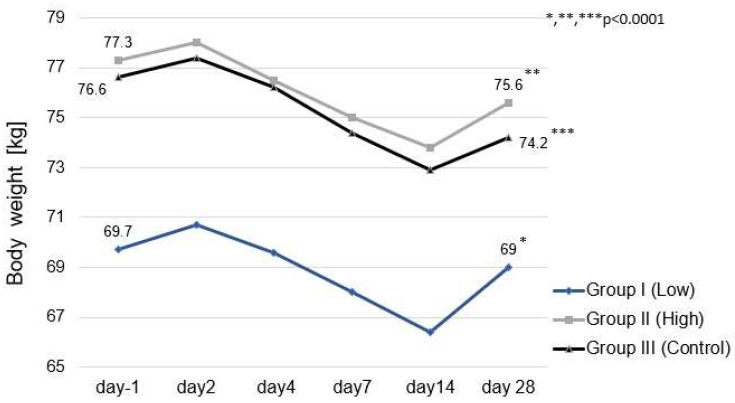
Body weight fluctuations before and after surgery in the group of patients taking low-osmotic (Group I) and high-osmotic (Group II) oral nutritional supplements in relation to the control group. The standard deviation (SD) did not exceed 16.8 in Group I, 14.8 in Group II, and 14.7 in Group III, respectively (not plotted).

**Figure 8:**
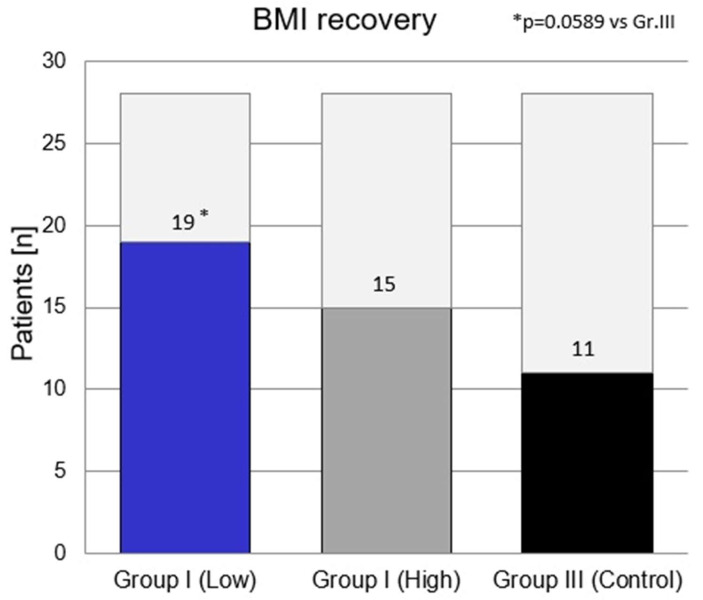
Number of patients whose body mass index (BMI) on Day 28 after surgery was the same or greater than the weight before surgery (Day −1).

Triglyceride levels rose after the procedure in all patients, peaking on the 7th postoperative day before beginning to decrease. Body weight decreased during the first fourteen days post-surgery but stabilized or returned to the preoperative baseline in the following two weeks (Figure 7). However, recovery to the preoperative BMI by the end of the observation period was more pronounced in the study groups receiving ONS support, particularly those with the low-osmotic formula (Figure 8).

## 4. Discussion

The results of this study confirm the metabolic benefits of initiating oral nutritional supplementation as early as possible following abdominal surgery. Recent research has demonstrated a significant correlation between perioperative malnutrition and the occurrence of postoperative complications, which notably influence long-term effects, particularly in oncological patients [5,19,20]. Improving the quality of surgical management, especially by minimizing perioperative complications, has been a key objective since the introduction of the ERAS protocol in the early 1990s. Subsequent studies have revealed that the long-term outcomes are not solely determined by technical proficiency or surgical experience but are also influenced by comprehensive prehabilitation and proper patient selection [5,21]. Preoperative analysis of patients’ risk factors enables the identification of individuals with an increased likelihood of postoperative complications.

According to ESPEN recommendations, factors such as age over 70 years, the extent of surgical trauma, exacerbation of comorbidities, and a diagnosis of malignancy are independent predictors of complications in surgical treatment [5]. Additionally, all these factors can individually alter a patient’s nutritional status and contribute to the development of metabolic deficiencies [4,22].

The demonstrated positive impact of supportive management before hospital admission laid the foundation for the concept of prehabilitation [1,4]. Current guidelines emphasize that even short-term intensive preoperative metabolic support, including oral supplementation, is beneficial for surgical patients and leads to improved long-term outcomes [4]. Moreover, it has been well established that undiagnosed or inadequately treated malnutrition prolongs hospital stays, increases healthcare costs, and elevates the risk of malnutrition recurrence [5,23,24].

From the standpoint of daily practice, it remains crucial to assess the actual absorption of nutrients contained in oral dietary supplements. While numerous studies have emphasized the beneficial effect of postoperative administration of ONSs, the commonly reported outcomes often rely on indirect evidence, such as prolonged length of stay (LOS), the surgical complication rate, or quality of life assessment [12,25]. Anthropometric parameters, as well as metabolic blood tests (e.g., total protein or albumin levels), may be helpful, but their reliability is limited due to factors such as surgery-induced stress, bowel obstruction, or perioperative fluids overload [26,27].

Surgery-induced trauma triggers a systemic inflammatory response, leading to the release of stress hormones and pro-inflammatory cytokines. Consequently, abdominal surgery, especially extensive procedures, may predispose patients to a catabolic state. Systemic inflammation results in the release of glucose, amino acids, and free fatty acids into the bloodstream [28,29,30]. Furthermore, postoperative recovery, including intestinal anastomosis and wound healing, demands increased energy and protein consumption.

In high-risk surgeries, i.e., complex procedures performed in older patients with coexisting severe and/or active chronic diseases such as diabetes, circulatory insufficiency, or inflammatory bowel diseases, nutritional deficiencies impair proper rehabilitation and contribute to a higher rate of complications [5,31]. Perioperative fluid retention, hydrothorax, anastomotic leakage, and surgical site infections are often rooted in albumin depletion and protein deficiency. Inadequate nutritional support results in a decrease in muscle mass, further exacerbating cachexia [32].

Analyses of changes in biochemical measurements in the postoperative period indicate a close correlation between the decline in blood lymphocytes, albumin, total protein, and cholesterol levels and surgery-induced trauma. Consequently, the interpretation of common metabolic data is controversial when attempting to establish actual nutritional deficiencies [28,33,34]. For instance, the perioperative interpretation of albumin levels remains challenging. While albumin typically serves as an indicator of malnutrition, it also functions as a negative heat shock protein, a marker of liver function, and a predictor of the risk of surgical complications [33,35,36,37]. Hence, controversy exists regarding the routine supplementation of albumin after surgical procedures and its reliability in assessing metabolic restitution [36,38,39,40,41].

We observed a marked decrease in albumin levels on Day 4, followed by an increase on Days 7 and 14, consistent with other studies. Considering all metabolic processes resulting from surgical treatment, albumin levels decline rapidly within the first two postoperative days due to hemodilution, fluid retention, and temporary liver dysfunction [38,42]. Notably, in both study groups, albumin levels returned to their preoperative value during the scheduled observation period, whereas in the control group, they remained decreased. This underscores the importance of nutritional supplementation following surgery.

Moreover, there are supportive opinions suggesting that albumin, as an osmotic coefficient, prevents fluid from shifting into extravascular and extracellular spaces, promoting better healing of anastomoses and surgical wounds [43,44]. On the contrary, other studies report that albumin administration does not lead to better outcomes and only increases hospital costs [38,42].

Following these considerations, it is suggested to measure the relative albumin level (∆ albumin) after surgery rather than its total value [40,41]. In our observations, the ∆ albumin level corresponded more closely to the severity of the inflammatory reaction and better reflected the tendency for postoperative regeneration in the two orally supplemented groups. Furthermore, the low-osmolality formula demonstrated a distinct advantage over the high-osmolality formula. Recent studies estimate that a decrease in the albumin level significantly correlates with both surgical and non-surgical postoperative complications [40,41]. An elevated CRP level with a simultaneous decrease in the albumin value is often observed in cases of anastomotic leakage or pneumonia [36,39,45].

As in the case of albumin, protein concentration followed a similar trajectory: a decrease in the initial days, followed by a subsequent increase. On Day 4 post-surgery, we observed a decrease in all three groups, with the high-osmolality group experiencing the smallest decrease. By Days 7 and 14, protein levels increased, with the low-osmolality group demonstrating an advantage over the high-osmolality group. However, on postoperative Day 14, protein levels in the low-osmolality group exceeded the baseline, while the high-osmotic ONS patients only returned to preoperative levels. In contrast, the control group ended up with a relative decline.

In the available literature, the total protein level is regarded as a reliable predictor of both postoperative malnutrition and surgical complications [46,47]. Moreover, based on serial monitoring of this marker, nutritional intervention can be effectively scheduled, and the effects of therapy can be strictly monitored [46,47].

The decision on preoperative nutritional supplementation should correspond to the extent of the procedure, considering factors such as the surgical approach (laparotomy vs laparoscopy) and technique (number and type of anastomoses). It is critical to account for procedure-related factors when deciding on the introduction of nutritional supplementation.

Surgery-related risk factors, typically nonmodifiable, closely correlate with secondary dysfunction of the gastrointestinal tract and intensify metabolic demands. Malnutrition stands out as a key predictor for early postoperative complications, such as anastomotic rupture, bowel obstruction, generalized edema, or respiratory failure. Nutritional deficiencies also impair tissue healing, consequently prolonging postoperative rehabilitation and delaying the implementation of further therapies. Nearly one-third of patients experience delays in adjuvant oncological procedures, such as chemotherapy or radiotherapy, due to weight loss following primary surgical treatment [48,49]. Similarly, preoperative malnutrition and prolonged postoperative fasting contribute to worse surgical outcomes [4,50].

The effectiveness of a nutritional intervention depends on the composition and amount of nutrients, as well as the osmolality of the mixture, coupled with the absorption capacity of the gastrointestinal tract [51,52]. Our study supports this thesis, demonstrating the significant advantage of low-osmotic over hyperosmotic formulas. The dose and frequency of administration should reflect the extent of intestinal resection, the efficiency of the remaining GI tract, and the feasibility of maintaining oral feeding after operation.

The positive impact of ONS on nutritional recovery appears to depend not only on the composition and dosage of the mixture but also on its ability to stimulate intestinal secretion and promote the growth of intraluminal microbiota. Osmolality—a derivative of the amount of dissolved substances, primarily the glucose concentration—plays a critical role. According to recent findings, osmolality significantly influences peristalsis and bowel transit, thereby facilitating nutrient absorption [25,53].

From a surgical standpoint, factors such as the type and number of intestinal anastomoses and the potential risk of secondary liver or pancreatic dysfunction are additional variables that affect the efficiency and rate of intestinal absorption [33,52]. A personalized nutrition plan should tailor the total caloric value, protein content, and the presence of dietary fiber to the individual patient’s needs [5,54]. For patients undergoing extensive surgical procedures, especially those with malignancies, their daily protein intake should be at least 1.4 g/kg of their body weight, and their total dietary energy intake should reach at least 25 kcal/kg.

Additionally, adequate supplementation with vitamins A, C, and E, along with microelements such as zinc, selenium, and copper, is critical for enhancing the patient’s recovery postoperatively [6,35,55].

One of the results of more protein intake is an upregulation glucagon-like peptide-1 (GLP-1), an incretin hormone synthesized in the intestines immediately after a meal. Oral diet as well as nutritional supplements filled with essential amino acids, especially leucine and whey protein, promote the secretion of GLP-1 and contribute indirectly to normalizing the blood sugar level and enhancing the feeling of satiety. On the other hand, an increase in the GLP-1 level inhibits peristalsis and delays gastric emptying. Furthermore, short-chain fatty acids (SCFAs) and soluble fiber components through microbiome fermentation also enhance GLP-1 activity. Therefore, the use of high-protein, low-calorie products enriched with SCFAs and fiber counteracts nutritional deficiencies while minimizing the risk of bloating and nausea [56,57,58].

Starvation, segmental resections, and gastrointestinal anastomoses, as well as stress associated with the procedure, contribute to disturbances in the gut microbiota. In addition, postoperative ileus and antibiotic therapy may facilitate colonization by pathogenic strains or promote the development of opportunistic infections [59]. The intake of ONSs also contributes to maintaining the intraluminal microbial balance, the intestinal barrier, and to restoring immune function. The supplementation of probiotics and prebiotics positively affects the proliferation of the intraluminal germs, which is crucial during prolonged hospital stays involving several days of antibiotics and admission to the intensive care unit [60,61].

This objective was central to our study. Both types of prescribed formulas provided protein and vitamins; however, we focused on comparing low- and high-osmolality formulas containing different amounts of protein and fat. The results indicate that a low-osmolality ONS delivers sufficient benefits, emphasizing that a larger amount of nutrients does not necessarily correlate with better metabolic effects. Simultaneously, oral diets should be individualized regarding consistency, volume, and any potential dietary restrictions [6,35].

Laboratory or anthropometric indicators commonly used to assess nutritional status before surgery may not reliably reflect a patient’s condition during the postoperative period. Parameters such as body weight, BMI, and triceps skinfold thickness lose sensitivity in the first days following surgery, with results often overestimated due to fluctuations in fluid volume and distribution. However, these indicators become more informative after the first postoperative week, as inflammatory responses to surgical trauma subside and fluid balance stabilizes. At this point, despite certain limitations, these measurements provide a valuable basis for planning nutritional interventions without significantly contributing to misinterpretation [33,55,62].

A particular challenge with these parameters is postoperative weight gain caused by fluid overload, often due to excessive fluid retention, circulatory failure, or sepsis [60,61,62]. This can lead to inaccuracies in nutritional assessments. To address this, nutritional plans should focus on lean body mass rather than total body weight [49].

To better address this issue, body composition analysis using bioelectrical impedance analysis (BIA) is recommended. This method measures the body’s total electrical resistance (passive and active) through surface electrodes connected to a detector, allowing for the determination of the fat-free body mass (FFM), fat mass (FM), and total body water (TBW). However, BIA has limitations in the postoperative period, including challenges with electrode placement, fluid imbalances, edema, and improper body alignment [63].

Additionally, Marano et al. propose that measuring the Handgrip Strength (HGS) in surgical patients offers a useful tool for diagnosing sarcopenia and assessing BMD. HGS also serves as a predictive factor for the LoS. Nevertheless, variations in dynamometer types, measurement protocols, patient positioning, and differences in height, gender, and age can undermine the consistency and validity of these measurements [64].

An increase in body weight due to fluid imbalances strongly correlates with a higher incidence of postoperative complications and longer hospital stays [24,34,55,62]. In line with these observations, our study recorded an increase in body weight on the second postoperative day, followed by a decline that reached its lowest point on Day 14. After this, body weight began to increase again. A similar pattern was observed across all study groups, including the control group.

### Limitations

This study has several limitations. The sample size of 84 patients may be insufficient to detect subtle differences or to generalize findings for diverse populations undergoing abdominal surgery. The follow-up period was limited to 28 days, leaving the long-term effects of ONS use, such as recovery and complication rates, unexplored. The study lacked population diversity in factors such as age, gender, comorbidities, and surgical procedures, which could influence metabolic responses and ONS efficacy. Potential confounding variables, including preoperative nutritional status, perioperative medications, comorbidities, socioeconomic determinants, and surgical techniques, were not fully controlled. The absence of blinding and randomization introduces potential bias, and the control group lacked supplementation, limiting comparisons to standard dietary protocols or placebo groups. The study’s specificity to abdominal surgery can restrict its generalizability to other surgical populations. Laboratory tests and body weight were prioritized for evaluating nutritional therapy, but incorporating anthropometric measures, electrical bioimpedance, or grip strength assessment to measure catabolism, sarcopenia, and fluid retention could enhance future protocols.

## 5. Conclusions

In summary, our study confirms the benefits of oral supplementation in patients undergoing abdominal surgery as early as possible. Both anthropometric and laboratory indicators provide valuable data for planning the nutritional intervention; simultaneously, it is crucial to note that within three to seven days post-operation, these parameters may be unreliable due to inflammatory reactions and fluid imbalance. Considering the insufficiency of intestinal absorption resulting from temporary obstruction, as well as changes in luminal microflora and the presence of intestinal anastomoses, the use of low-osmotic ONSs is characterized by better tolerance, and therefore, it is easier to use in everyday practice. Dietary supplements with lower osmolality, especially with a limited amount of glucose but with a high protein concentration, seem to be the best choice for surgical patients. However, the final decision should consider patients’ metabolic condition and the range of nutritional support.

## Figures and Tables

**Table 1:** Profile of low- and high-osmotic formulas used in the study.

	Low-Osmotic ONS (per 100 mL)	High-Osmotic ONS Content (per 100 mL)
Osmolarity (mOsmol/L)	290	570
Energy value (kJ/kcal)	627/150	1029/245
Fat (g)	5.4	9.6
Saturated fatty acids	2.4	0.86
Protein (g)	9.4	14.6
Fiber (g)	0	0
Vitamins	Yes	Yes
Microelements	Yes	Yes

**Table 2:** Parameters and time frame of evaluation used in the study.

Parameter	Day −1	Day 4	Day 7	Day 14	Day 28
Lymphocytes	x	x	x	x	-
Triglycerides	x	x	x	x	-
Total cholesterol	x	x	x	x	-
Albumin	x	x	x	x	-
Total protein	x	x	x	x	-
Body weight	x	-	-	x	x

x: parameter evaluated; -: parameter not evaluated.

**Table 3:** The basic characteristics of the patient cohort.

	Group I (Low-Osmotic ONS)	Group II (High-Osmotic ONS)	Group III (Control)
Age (range/mean)	23–85/59.4	28–81/60.1	27–84/58.3
Sex (f/m)	19/9	18/10	15/13

**Table 4:** Underlying pathology and type of performed intestinal anastomosis in the study.

Underlying Pathology	Number of Patients, *n* (%)
Colorectal cancer:	
Rectum	21 (25)
Large bowel	30 (35.7)
Non-oncological indications for surgery:	
Restoration of GI tract continuity	14 (16.7)
Complicated diverticular disease	7 (8.3)
Adhesiolysis with segmental small bowel resection	5 (6)
Low-output enterocutaneous fistulas	3 (3.6)
Type of Intestinal Anastomosis	Number of Patients, *n* (%)
Small bowel–small bowel	25 (29.8)
Small bowel–large bowel	10 (11.9)
Large bowel–rectum	44 (52.4)
Large bowel–large bowel	9 (10.7)

## Data Availability

The datasets used and/or analyzed during the current study are available from the corresponding author on reasonable request.

## Abbreviations

The following abbreviations are used in this manuscript:

ANOVA	analysis of variance
ASA	American Society of Anesthesiologists
BIA	bioelectrical impedance analysis
BMI	body mass index
ERAS	enhanced recovery after surgery
ESPEN	The European Society of Parenteral and Enteral Nutrition
FFM	fat-free body mass
FM	fat mass
GI	gastrointestinal
GLIM	Global Leadership Initiative on Malnutrition
HGS	handgrip strength
LOS	length of stay
NICE	National Institute for Health and Care Excellence
ONS	oral nutritional supplements
SD	standard deviation
TBW	total body water
